# The Epidemiology and Outcomes of Acute Kidney Injury in Patients With Chronic Kidney Disease: A Single-Center Retrospective Cohort Study

**DOI:** 10.1155/anrp/6657933

**Published:** 2025-05-27

**Authors:** Shunsuke Oura, Marie Okada, Ryo Miyashita, Shuji Yamamoto

**Affiliations:** ^1^Department of Anesthesiology, Sapporo Higashi Tokushukai Hospital, Sapporo, Hokkaido, Japan; ^2^Department of Anesthesiology, Obihiro-Kosei General Hospital, Obihiro, Hokkaido, Japan

**Keywords:** acute kidney injury, chronic kidney disease, estimated glomerular filtration rate, ischemic heart disease

## Abstract

**Background:** Previous studies have highlighted the association between chronic kidney disease (CKD) and the increased incidence of postoperative acute kidney injury (AKI). However, the risk factor and incidence of postoperative AKI in patients with CKD undergoing elective surgery remained unclear. This retrospective study aimed to evaluate the perioperative predictors of postoperative AKI in patients with CKD.

**Methods:** Data from 137 patients with CKD, defined by an estimated glomerular filtration rate (eGFR) < 60 mL/min/1.73 m^2^, who underwent elective surgery under general or regional anesthesia between November 2018 and October 2023, were retrospectively reviewed. Patients were placed on a peripheral arterial catheter, and dual arterial blood gas analysis was performed within 30 min before and after surgery. Patients undergoing cardiac surgery, emergency surgery, preoperative renal replacement therapy, as well as those with missing data, were excluded from the study. Both general and local anesthesia modalities were included in the analysis.

**Results:** The incidence of postoperative AKI was 24%. All cases of AKI were classified as Stage 1. Preoperative ischemic heart disease (odds ratio: 2.660, 95% CI: 1.16–6.10, *p*=0.00207) and lower eGFR (odds ratio: 0.947, 95% CI: 0.915–0.980, *p*=0.00181) were associated with increased risk of postoperative AKI. More patients who developed postoperative AKI converted to maintained dialysis compared to patients who did not develop AKI (15% vs. 2.1%, *p*=0.0021).

**Conclusions:** History of ischemic heart disease and preoperative lower eGFR and may serve as risk factors for postoperative AKI in CKD patients.

## 1. Introduction

Postoperative acute kidney injury (AKI) has been reported to occur in 1%–31% of cases depending on the type of surgery and preoperative patient status [1–3]. In general cohort with varying degree of renal function, identified risk factors for postoperative AKI have reported to include male sex, age > 50 years, body mass index (BMI), diabetes mellitus, hypertension, ascites, heart failure, emergency surgery, intraperitoneal surgery, polypharmacy, use of an angiotensin-converting enzyme (i.e., “ACE”) inhibitor or angiotensin receptor blocker (i.e., “ARB”), and increased American Society of Anesthesiologists physical status (ASA–PS) scores [1, 4, 5]. Previous investigations also have highlighted the association between chronic kidney disease (CKD) and an increased incidence of postoperative AKI [5, 6]. The presence of CKD was associated with a high incidence of AKI after total joint arthroplasty [7]. However, the risk factor of postoperative AKI in patients with reduced preoperative kidney function has not been fully investigated. This study aimed to elucidate the preoperative, intraoperative, and postoperative risk factors associated with postoperative AKI in patients with CKD.

## 2. Methods

### 2.1. Ethical Considerations

This study was conducted according to the Declaration of Helsinki, with approval of the Local Ethics Committee of Obihiro Kosei Hospital, Hokkaido, Japan (Approval number: 2023-111), and the requirement for informed consent was waived. Instead, an opt-out option had been published on the internet homepage, which allowed patients to decline their participation in the study.

### 2.2. Study Design and Patients

**Figure 1:**
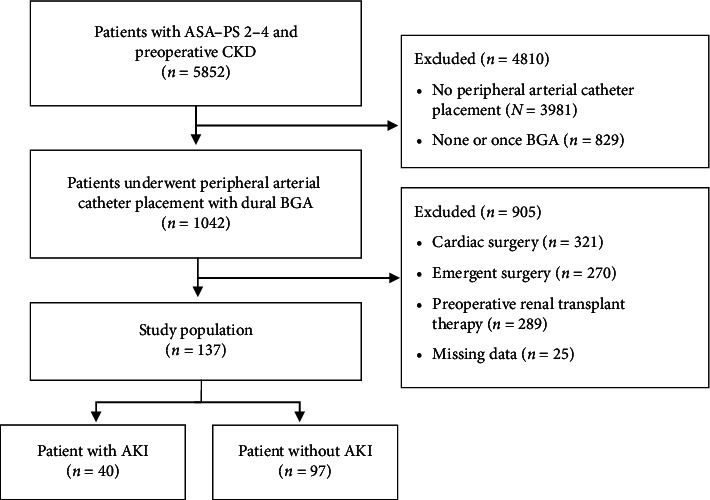
Flowchart of the patient groups included in the study. AKI: acute kidney injury; ASA–PS: American Society of Anesthesiologists physical status; BGA: blood gas analysis; CKD: chronic kidney disease.

Data of adult patients from November 2018 to October 2023 at the institution with an ASA–PS score 2–4 and preoperative CKD with an estimated glomerular filtration rate (eGFR) < 60 mL/min/1.73 m^2^ were retrospectively analyzed.  Figure 1 shows the patient's selection flowchart. To evaluate the intraoperative risk factor of AKI, patients who underwent peripheral arterial catheter placement with dual arterial BGA performed within 30 min before and after surgery were selected in the study (*n* = 1042). Under this condition, patients undergoing cardiac surgery (*n* = 321), emergency surgery (*n* = 270), or preoperative renal replacement therapy (*n* = 289), as well as those with missing data (13 cases with missing intraoperative urine volume and 12 cases with unknown preoperative history), were excluded.

### 2.3. Perioperative Anesthetic Management

General anesthesia was induced using propofol, fentanyl, and remifentanil, followed by tracheal intubation with rocuronium. Anesthesia was maintained using propofol and remifentanil-based total intravenous anesthesia or inhalation anesthesia with sevoflurane/desflurane and remifentanil, according to the preferences of the anesthetists and/or surgeons. Epidural anesthesia or a peripheral nerve block was also administered with or without general anesthesia.

### 2.4. Outcome Measurements

AKI was diagnosed based on the serum creatinine (sCr) levels component of the Kidney Disease Improving Global Outcomes (KDIGO) criteria [8]. Baseline (i.e., preoperative) sCr levels were determined based on values measured within 1 month before surgery. AKI was defined as follows: Stage 1 (change in sCr level ≥ 0.3 mg/dL within 48 h, or a rise in sCr level 1.5–1.9 times within 7 days relative to the baseline sCr level), Stage 2 (rise in sCr level 2–2.9 times within 7 days, relative to the baseline sCr level), and Stage 3 (rise in sCr level 3 times within 7 days, relative to the baseline sCr level). Urine output on postoperative Day (POD) 1 was obtained from nursing medical records. Laboratory investigation results obtained within 1 month before surgery were used in data analysis. Baseline and perioperative data for all patients were obtained from anesthesia charts and medical records. Individual information was anonymized during data collection and no personal information was disclosed.

### 2.5. Sample Size and Statistical Analysis

Continuous and categorical variables were compared between the AKI and non-AKI groups. Categorical variables were analyzed using Fisher's exact test, while differences between the means of the groups were assessed using the Mann–Whitney *U* test. Categorical variables are expressed as numerical values, while continuous variables are expressed as mean ± standard deviation (SD). Differences with *p* < 0.05 were statistically significant. These statistical analyses were performed using EZR [9] (Saitama Medical Center, Jichi Medical University, Saitama, Japan), which is a graphical user interface for R (R Foundation for Statistical Computing, Vienna, Austria). More precisely, it is a modified version of the R Commander designed to add statistical functions frequently used in biostatistics.

## 3. Results

Based on the inclusion and exclusion criteria described in the Methods, 137 patients were included in this study (Figure 1). The incidence of AKI on PODs 1–7, based on the KDIGO criteria, was 29.2% (40/137). All cases of AKI were classified as Stage 1. The percentage of patients with preoperative CKD Stage 4 or Stage 5 was as high as 28.4% (39/137). Comparison of preoperative patient characteristics between the AKI and non-AKI groups revealed no significant differences in age, BMI, sex, ASA–PS, and smoking history (Table 1). The prevalence of ischemic heart disease was significantly higher in the AKI group (50% versus 30%, *p*=0.032) (Table 1). According to preoperative laboratory investigations, mean eGFR levels were significantly lower in the AKI group than in the non-AKI group (31.9 ± 13.1 vs. 38.8 ± 11.9, *p*=0.004) and the percentage of preoperative CKD Stage 4 and Stage 5 was higher in the AKI group (45% versus 21.6%, *p*=0.012) (Table 1). There were no statistically significant differences between other laboratory data, preoperative medical history, and AKI progression (*p* > 0.05) (Table 1).

The ratio of surgical types had no statistical difference between the AKI and non-AKI groups (*p* > 0.05) (Table 2). The overall ratio of general and/or local anesthesia modalities differed between the AKI and non-AKI groups. The ratio of patients who developed AKI was significantly higher in the general anesthesia and epidural anesthesia groups (*p*=0.04) and lower in the general anesthesia and peripheral nerve block groups (*p*=0.002) (Table 2). The intraoperative and postoperative characteristics had no statistical difference between the AKI and non-AKI groups (*p* > 0.05) (Table 3). Intraoperative dialysis was not performed, but glucose-insulin therapy was used to correct hyperkalemia in the AKI group (Table 3). Preoperative/postoperative differences in BGA are summarized in Table 4. Notably, intraoperative acidosis statistically progressed (ΔpH) in both the AKI (*p*=0.035) and non-AKI groups (*p* < 0.001), but only with slight changes. Intraoperative parameters based on BGA were not significantly different between the two groups (*p* > 0.05).

Postoperative clinical complications are summarized in Table 5. The postoperative renal function in the AKI group tended to deteriorate without statistical significance (*p*=0.056). More patients in the AKI group required maintenance dialysis compared to the non-AKI group (15% vs. 2.1%, *p*=0.0021). In all cases of postoperative AKI, eGFR returned to the same preoperative level within 7 days after surgery. Maintenance dialysis was introduced one month to one year after surgery. The association with postoperative AKI was evaluated through multiple logistic regression analysis, with age, ischemic heart disease, eGFR and ASA–PS as independent variables (Table 6). Notably, ischemic heart disease (odds ratio: 2.660, 95% CI: 1.16–6.10, *p*=0.00207) and lower eGFR (odds ratio: 0.947, 95% CI: 0.915–0.98, *p*=0.00181) were associated with increased risk of postoperative AKI.

## 4. Discussion

Results of this single-center, retrospective study revealed that the incidence of postoperative AKI in patients with CKD was 24%. A previous study revealed that the incidence of postoperative AKI after noncardiac surgery was 1% in patients with normal renal function [1] or 6.3% in patients with various renal impairments [2]. Conversely, the incidence of AKI was reported to be 31% following orthopedic trauma surgery [3] or following total joint arthroplasty in CKD patients [7], which means that the incidence of AKI varies substantially depending on patient condition and surgical types. Notably, all cases of AKI in the present study were classified as Stage 1. This finding may reflect the impact of meticulous perioperative management by anesthesiologists and other healthcare personnel, potentially mitigating the severity of AKI. The exclusion of patients undergoing maintained dialysis may also have contributed to the absence of more severe AKI (Stage 2 or Stage 3). In our study, the incidence of AKI was relatively high compared to the previous study focused on noncardiac surgery, which might explain that the impaired kidney is less capable of undergoing complete adaptive repair after an acute stress from elective surgery. CKD is associated with pre-existing microvascular and tubular injury, which might increase the susceptibility to further acute insults during surgery and the incidence of AKI [10]. Previous research has identified risk factors for postoperative AKI, include male sex, age > 50 years, diabetes mellitus, hypertension, ascites, heart failure, emergency surgery, intraperitoneal surgery, polypharmacy, use of an “ACE” inhibitor or “ARB,” and increased ASA–PS scores [4, 5]. Patients with CKD and/or diabetes mellitus are at particularly high risk for postoperative AKI [4]. In CKD patients, the incidence of AKI after total joint arthroplasty was reported to be high [7]. However, the risk factor of postoperative AKI in patients with CKD has not been fully investigated. In our study, we focused on patients with impaired renal function who were not undergoing hemodialysis and investigated the risk factors for postoperative AKI. We found that preoperative lower eGFR and a history of ischemic heart disease were significant risk factors for postoperative AKI in CKD patients, consistent with factors identified in the previous research among the general cohort [5]. However, several factors (i.e., age, diabetes mellitus, and hypertension) were not statistically associated with the risk factor of postoperative AKI. One possible explanation is that diabetes mellitus, hypertension, and kidney dysfunction are related to microvascular dysfunction [11], which might not add further risk to CKD patients who already have vascular dysfunction.

Previous studies have shown that perioperative metabolic acidosis in patients undergoing craniotomies or infants undergoing open laparotomies is associated with an increased incidence of postoperative AKI [12, 13]. In addition, a previous study reported that intraoperative metabolic acidosis occurs more frequently during renal transplant surgery in patients with CKD without maintenance hemodialysis than in those undergoing maintenance hemodialysis [14]. Therefore, we also investigated whether intraoperative BGA-based assessment of metabolic acidosis could predict postoperative AKI. Our data demonstrated that metabolic acidosis progressed during surgery in both groups, which is consistent with findings reported in a previous study [15]. However, there was no significant association between perioperative metabolic acidosis and the occurrence of postoperative AKI. One possible explanation is that short-term metabolic acidosis during surgery can be compensated by the kidneys with impaired function. Nevertheless, some patients in the AKI group had deteriorated kidney function and required maintenance dialysis, suggesting that metabolic acidosis may not fully recover during surgery or the postoperative period because of impaired renal function and permanent damage to the impaired kidney [16]. Another contributing factor may be that anesthesiologists made efforts to prevent progression to metabolic acidosis using sodium bicarbonate and/or goal-oriented fluid therapy [17], maintaining a mean blood pressure above 65 mmHg during surgery [18]. While some evidence suggests a potential benefit of perioperative sodium bicarbonate infusion in reducing the incidence of AKI, especially in cardiac surgery [19], other studies have reported potentially harmful renal effects associated with its use [20]. In addition, some studies have reported that goal-directed fluid therapy was not associated with an improved rate of postoperative AKI [21]. Further studies are required to fully elucidate these mechanisms.

Although a greater proportion of patients in the AKI group required maintenance dialysis compared to the non-AKI group, no significant difference in 1-year mortality was observed between the two groups (Table 5). It is not unclear whether AKI increases mortality from the underlying disease, although previous studies have established that AKI is associated with increased long-term mortality and progression of renal dysfunction [22], Prior research has indicated that neuraxial anesthesia tends to decrease the incidence of postoperative AKI compared to general anesthesia in patients undergoing total knee arthroplasty [23]. In our study, there were no significant differences in the distribution of anesthesia techniques between the AKI and non-AKI groups. However, the number of patients who underwent surgery exclusively under neuraxial anesthesia was limited, thus precluding rigorous statistical analysis.

This present study had some limitations. First, its single-center, retrospective design and limited number of cases mean our results may not be generalizable. Although our institution has various clinical departments, the surgical departments in this investigation are heterogeneous, and there were no cases of gynecological surgery. To examine intraoperative BGA data, we selected the patients who underwent peripheral arterial catheter placement, which resulted in reducing the number of cases to be analyzed to more than 80%. Despite meticulous data collection from anesthesia charts and medical records, not all data were extracted, These factors limited the number of cases (Figure 1) and had a potential impact on the results. Second, postoperative management varied depending on the instructions of each clinical department, which may have influenced the occurrence of postoperative AKI. Third, postoperative urine output was not used as a criterion in the KDIGO classification in this study because it was not adequately measured in the wards. This may have led to an underestimation of the incidence of postoperative AKI. Fourth, CKD is also classified based on cause, GFR category, and albuminuria category, abbreviated as CGA [24] This could enhance the clinical significance. However, few patients were evaluated for preoperative urinary albumin, which was not sufficient for statistical evaluation and interpretation. Fifth, previous research has indicated that a mean arterial pressure (MAP) below 65 mmHg is closely associated with an increased risk for postoperative AKI in noncardiac operations [18]. Our data demonstrated that the nadir MAP in both groups was below 65 mmHg and the cumulative time of MAP was over 30 min, which may have affected the incidence of postoperative AKI, although there were no statistically significant differences. Finally, although ischemic heart disease and lower eGFR were identified as risk factors for postoperative AKI, the small number of limited cohorts made it difficult to generalize these findings. Moreover, unforeseen variables not captured in this study may also contribute to postoperative AKI risk, despite our efforts to select variables based on previous research [1, 4, 5].

Despite these limitations, our study is novel in incorporating intraoperative blood gas analysis to investigate postoperative AKI risk in patients with impaired renal function. Further large-scale, prospective studies are warranted to better elucidate the risk factors and underlying mechanisms of postoperative AKI in this vulnerable population.

## 5. Conclusions

In CKD patients who underwent elective surgery, the incidence of postoperative AKI was 24%, which was relatively high compared to that in the general cohort. Preoperative lower eGFR and a history of ischemic heart disease may serve as risk factors for postoperative AKI in CKD patients. Our findings bear significance for perioperative management and preoperative assessment aimed at preventing the postoperative renal failure and fostering increased awareness regarding meticulous anesthetic management, such as stricter intraoperative blood pressure control and the selection of analgesics with minimal nephrotoxic potential for patients with predisposing risk factors.

## Figures and Tables

**Table 1:** Demographic characteristics of AKI and non-AKI groups.

Demographic data	AKI (*n* = 40)	Non-AKI (*n* = 97)	*p* value
Age (years)	74.6 ± 8.0	72.1 ± 9.9	0.231
BMI (kg/m^2^)	24.5 ± 3.9	24.4 ± 4.5	0.795
Male sex	31 (77.5)	76 (78.4)	> 0.99
ASA–PS 2	19 (47.5)	45 (46.4)	> 0.99
ASA–PS 3	19 (47.5)	46 (47.4)	> 0.99
ASA–PS 4	2 (5)	6 (6.2)	> 0.99
CKD Stages 4 and 5	18 (45)	17 (21.6)	0.012^∗^

*Complications*			
Past and current smoker	32 (73.3)	68 (70.1)	0.293
Diabetes mellitus	12 (30)	39 (40.2)	0.332
Hypertension	29 (72.5)	73 (75.2)	0.830
Ischemic heart disease	20 (50)	29 (29.9)	0.032^∗^
Cerebrovascular disease	10 (25)	18 (18.6)	0.485
Pulmonary disease	13 (32.5)	31 (32)	> 0.99

*Preoperative laboratory data*			
Hemoglobin (g/dL)	11.4 ± 1.8	12.0 ± 2.3	0.119
AST (IU/L)	26.3 ± 14.2	24.4 ± 11.7	0.851
ALT (IU/L)	24.1 ± 22.4	20 ± 12.1	0.953
Albumin (g/dL)	3.4 ± 0.6	3.7 ± 1.4	0.080
eGFR (mL/min/1.73 m^2^)	31.9 ± 13.1	38.8 ± 11.9	0.004^∗^

*Note:* Data were expressed as mean ± standard deviation, or the number of patients' percentages were shown in parentheses (). ^∗^Statistically significant *p* values (< 0.05).

Abbreviations: ALT, alanine aminotransferase; ASA–PS, American Society of Anesthesiologists physical status; AST, aspartate aminotransferase; BMI, body mass index; BUN; blood urea nitrogen; eGFR, estimated glomerular filtration rate.

**Table 2:** Surgical type and anesthesia methods of the AKI and non-AKI groups.

Surgical type	AKI (*n* = 40)	Non-AKI (*n* = 97)	*p* value
General surgery	22 (55)	45 (46.4)	0.453
Plastic surgery	0 (0)	1 (1)	> 0.99
Otolaryngological surgery	2 (5)	3 (3.1)	0.629
Orthopedic surgery	5 (12.5)	16 (16.5)	0.795
Brain surgery	2 (5)	6 (6.2)	> 0.99
Urological surgery	9 (2.5)	26 (26.8)	0.671

*Anesthesia methods*			
GA	8 (20)	18 (18.6)	0.815
GA and Epi	28 (70)	49 (50.5)	0.040^∗^
GA and PNB	2 (5)	27 (27.8)	0.002^∗^
Epi and/or Spi	2 (5)	3 (3.1)	0.629

*Note:* Data were expressed as mean ± standard deviation, or the number of patients' percentages were shown in parentheses (). ^∗^Statistically significant *p* values (< 0.05).

Abbreviations: Epi, epidural anesthesia; GA, general anesthesia; PNB, peripheral nerve block; Spi, spinal anesthesia.

**Table 3:** Intraoperative and postoperative characteristics of the AKI and non-AKI groups.

	AKI (*n* = 40)	Non-AKI (*n* = 97)	*p* value
Use of a vasopressor	35 (87.5)	87 (90)	0.766
RBC and/or FFP transfusion	4 (10)	6 (6.2)	0.477
Infused crystalloid (mL)	1801 ± 796	1727 ± 976	0.282
Urine output (mL)	226 ± 216	283 ± 317	0.634
Blood loss (mL)	137 ± 200	169 ± 282	0.437
Input–output (mL)	1472 ± 727	1332 ± 910	0.095
The lowest MAP (mmHg)	54.9 ± 9.6	57.4 ± 10.8	0.260
Intraoperative cumulative time of MAP < 65 mmHg (minutes)	38.2 ± 33.8	31.4 ± 35.7	0.163
Operation time (minutes)	242 ± 124	224 ± 126	0.357
Glucose-insulin therapy	2 (5)	0 (0)	0.151

*Note:* Data were expressed as mean ± standard deviation, or the number of patients' percentages were shown in parentheses ().

Abbreviations: FFP, fresh frozen plasma; MAP, mean arterial pressure; RBC, red blood cell.

**Table 4:** BGA of the AKI and non-AKI groups.

	AKI (*n* = 40)	Non-AKI (*n* = 97)	*p* value
ΔpH	−0.016 ± 0.048	−0.024 ± 0.051	0.724
ΔPaCO_2_	0.87 ± 5.2	0.96 ± 5.9	0.932
ΔBE	−0.795 ± 1.88	−0.579 ± 2.39	0.529
ΔHCO_3_	−0.58 ± 1.763	−0.185 ± 2.37	0.424
ΔPotassium	0.1423 ± 0.545	0.233 ± 0.448	0.476
ΔLac	2.645 ± 3.434	2.592 ± 4.373	0.397

*Note:* Data were expressed as mean ± standard deviation. Δ, postoperative–preoperative data.

Abbreviations: BE, base excess; Lac, lactase.

**Table 5:** Postoperative clinical complications of the AKI and non-AKI groups.

	AKI (*n* = 40)	Non-AKI (*n* = 97)	*p* value
Cardiovascular complication	3 (7.5)	8 (8.2)	> 0.99
Pulmonary complication	13 (32.5)	31 (32.0)	> 0.99
Deep vein/thrombosis/pulmonary thromboembolism	1 (2.5)	2 (2.1)	> 0.99
Delirium	4 (10)	5 (5.2)	0.448
Neurological complication	1 (2.5)	4 (4.1)	> 0.99
Surgical site infection	1 (2.5)	6 (6.2)	0.673
Gastrointestinal complication	6 (15)	8 (8.2)	0.234
Intensive care unit admission	2 (5)	9 (9.3)	0.509
Worsening of postoperative eGFR over 30 days	29 (72.5)	52 (53.6)	0.056
Maintained dialysis	6 (15)	2 (2.1)	0.002^∗^
Death at 30 days	1 (2.5)	0 (0)	0.292
Death at 1 year	8 (20)	11 (11.3)	0.187

*Note:* Data were expressed as mean ± standard deviation, or the number of patients' percentages were shown in parentheses (). ^∗^Statistically significant *p* values (< 0.05).

**Table 6:** Multivariable indicators of postoperative AKI development.

	*p* value	OR	95% CI
Age	0.289	1.020	0.979–1.07
Ischemic heart disease	0.021^∗^	2.660	1.16–6.10
eGFR	0.002^∗^	0.947	0.915–0.98
ASA–PS	0.138	0.573	0.274–1.20

*Note:* ^∗^Statistically significant *p* values (< 0.05).

## Data Availability

The data of this study are available from the corresponding author upon reasonable request. The data are not publicly available due to privacy or ethical restrictions.
